# Supplementary figures and images for: First report of fatty acids in Mimosadiplotricha bee pollen with in vitro lipase inhibitory activity
Source: PeerJ. 2022 Jan 3;10:e12722. doi: 10.7717/peerj.12722 (PMC8734463; doi:10.7717/peerj.12722)

NAME of F2-1

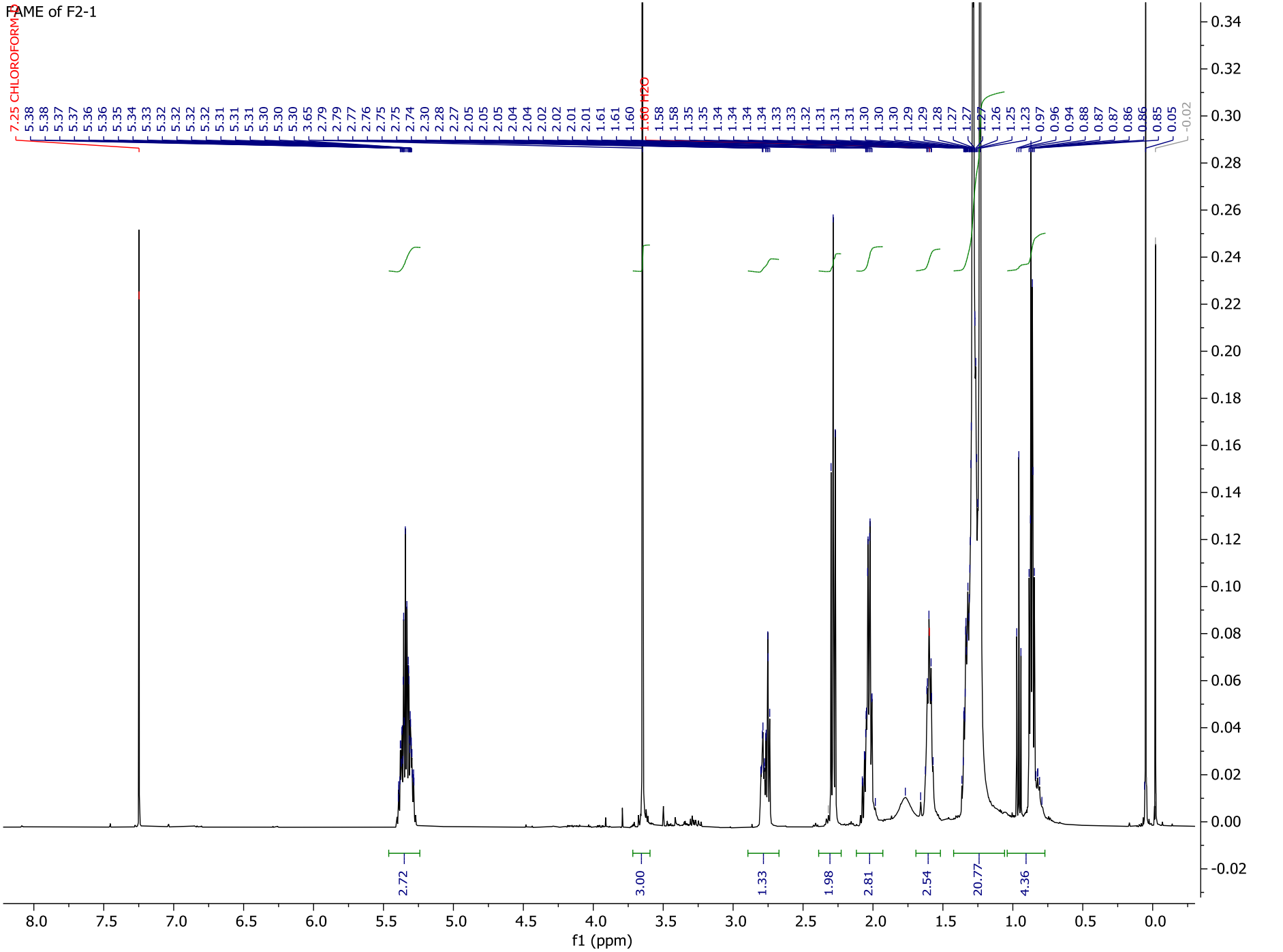

Supplement: Supplemental Information 3 [file peerj-10-12722-s003.pdf]

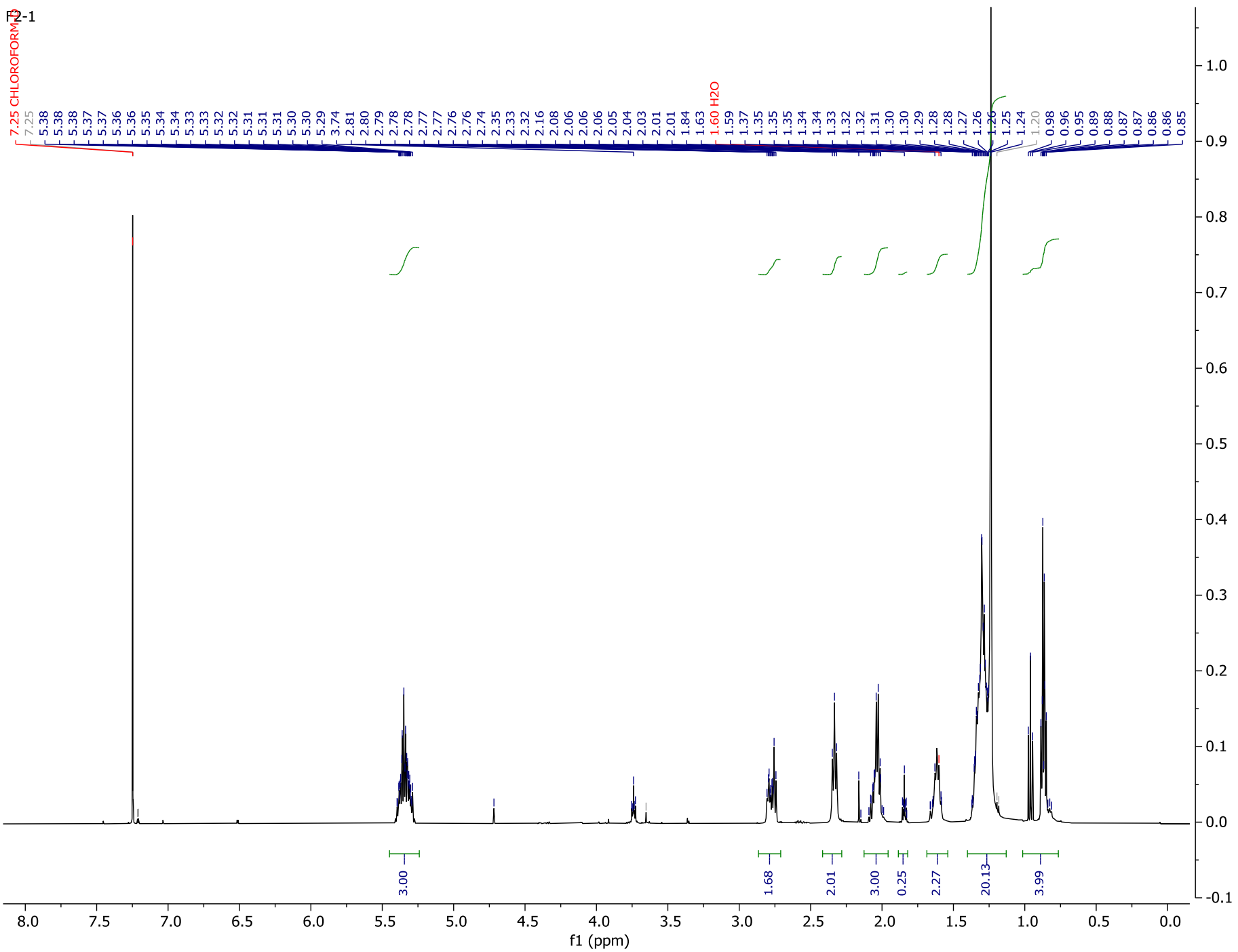

Supplement: Supplemental Information 4 [file peerj-10-12722-s004.pdf]

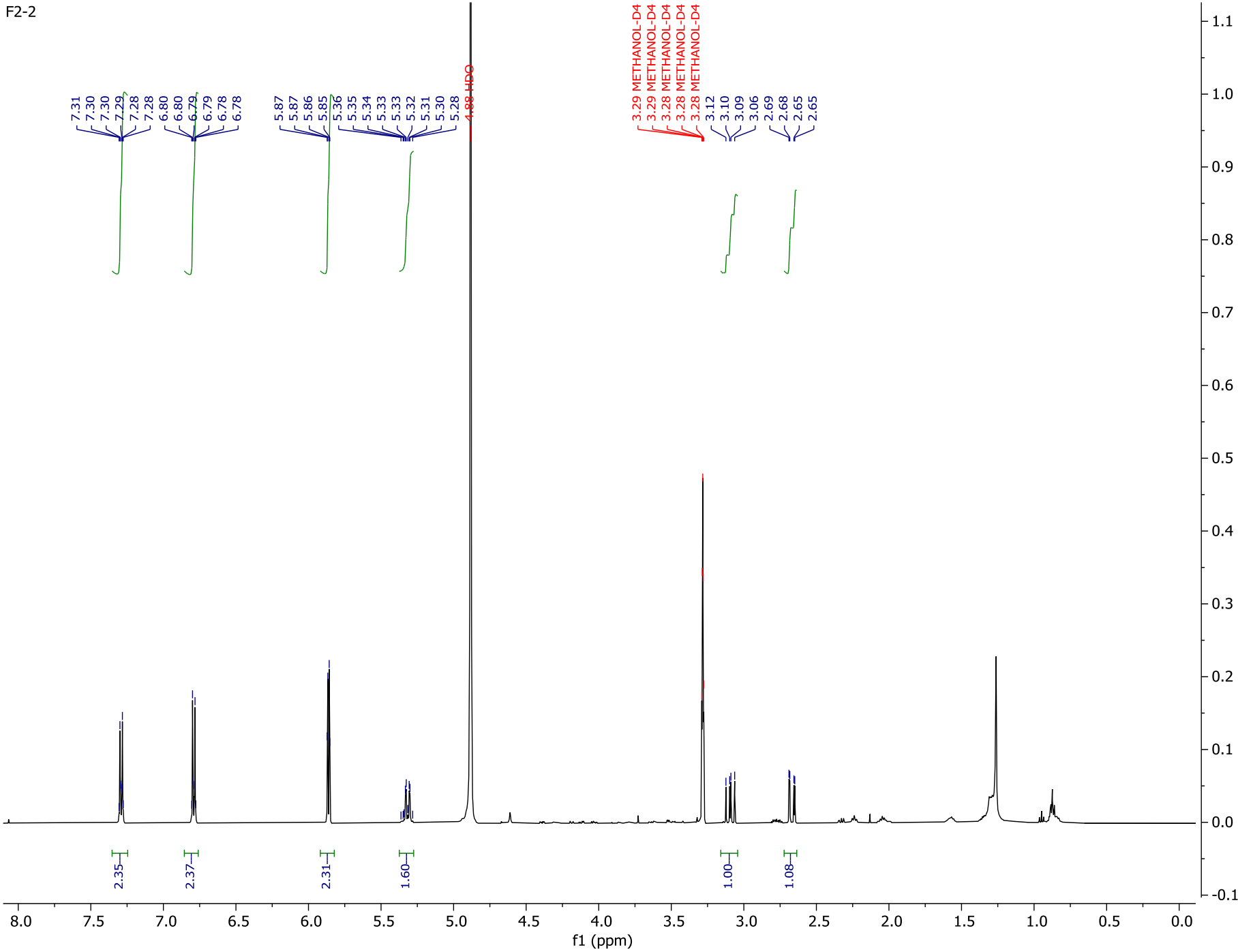

Supplement: Supplemental Information 5 [file peerj-10-12722-s005.pdf]

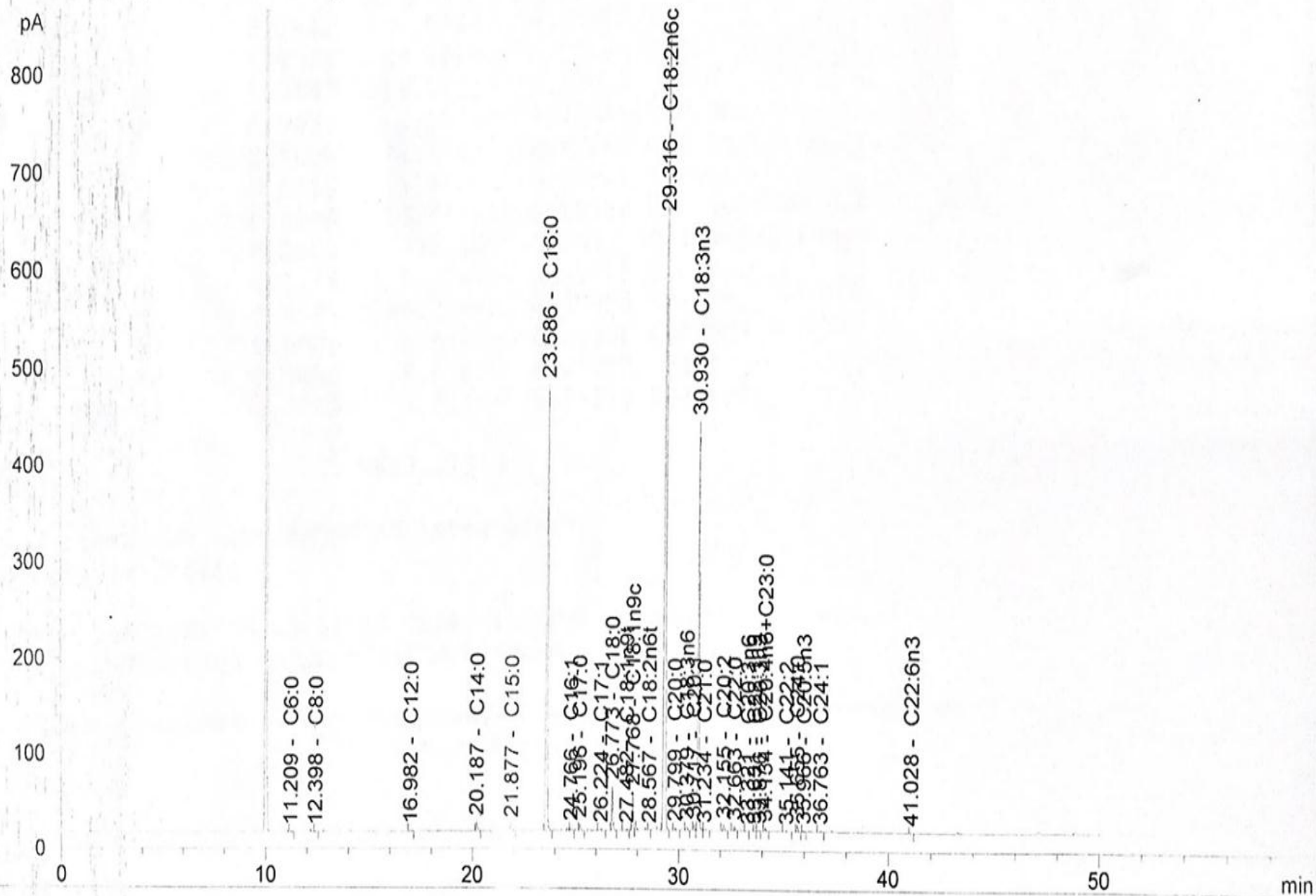

Supplement: Supplemental Information 6 [file peerj-10-12722-s006.pdf]
